# The Beneficial Effects of Cognitive Training With Simple Calculation and Reading Aloud (SCRA) in the Elderly Postoperative Population: A Pilot Randomized Controlled Trial

**DOI:** 10.3389/fnagi.2018.00068

**Published:** 2018-03-28

**Authors:** Kay Kulason, Rui Nouchi, Yasushi Hoshikawa, Masafumi Noda, Yoshinori Okada, Ryuta Kawashima

**Affiliations:** ^1^Department of Advanced Brain Science, Institute of Development, Aging and Cancer, Tohoku University, Sendai, Japan; ^2^New York Institute of Technology College of Osteopathic Medicine, New York, NY, United States; ^3^Creative Interdisciplinary Research Division, Frontier Research Institute for Interdisciplinary Science, Tohoku University, Sendai, Japan; ^4^Human and Social Response Research Division, International Research Institute of Disaster Science, Tohoku University, Sendai, Japan; ^5^Department of Thoracic Surgery, Fujita Health University School of Medicine, Toyoake, Japan; ^6^Department of Thoracic Surgery, Institute of Development, Aging and Cancer, Tohoku University, Sendai, Japan; ^7^Division of Developmental Cognitive Neuroscience, Institute of Development, Aging and Cancer, Tohoku University, Sendai, Japan

**Keywords:** cognitive training, FAB, depression, QOL, thoracic surgery

## Abstract

**Background:** There has been little research conducted regarding cognitive treatments for the elderly postsurgical population. Patients aged ≥60 years have an increased risk of postoperative cognitive decline, a condition in which cognitive functions are negatively affected. This cognitive decline can lead to a decline in quality of life. In order to maintain a high quality of life, the elderly postsurgical population may benefit from treatment to maintain and/or improve their cognitive functions. This pilot study investigates the effect of simple calculation and reading aloud (SCRA) cognitive training in elderly Japanese postsurgical patients.

**Methods:** Elderly patients undergoing non-cardiovascular thoracic surgery under general anesthesia were recruited (*n* = 12). Subjects were randomly divided into two groups—one that receives 12 weeks of SCRA intervention, and a waitlisted control group. Before and after the intervention, we measured cognitive function [Mini-Mental Status Exam-Japanese (MMSE-J), Frontal Assessment Battery (FAB), computerized Cogstate Brief Battery (CBB)] and emotional state [General Health Questionnaire-12 (GHQ-12), Geriatric Depression Scale (GDS), Quality of Life Scale-5 (QOL-5)].

**Results:** Group difference analyses using ANCOVA with permutation test showed that the intervention SCRA group had a significant improvement in FAB motor programming sub-score, GDS, and QOL-5 compared to the control group. Within-group analyses using Wilcoxon signed-rank test to compare baseline and follow-up showed that the SCRA intervention group total FAB scores, FAB motor programming sub-scores, and QOL-5 scores were significantly improved.

**Discussion:** This pilot study showed that there are important implications for the beneficial effects of SCRA intervention on cognitive function and emotional state in the postoperative elderly population; however, further investigations are necessary to reach any conclusions.

**Trial registration:** This study was registered with the University Hospital Medical Information Network (UMIN) Clinical Trial Registry (UMIN000019832).

## Introduction

### Background and rationale

Cognitive changes in elderly patients after surgery have been reported since the 1950s, and anesthesia has often been hypothesized to be the cause. In a review of 1,193 aged patients who underwent surgery with general anesthesia, Bedford concluded that general anesthetics, hypotension, and cognitive changes were related, and that “operations on elderly people should be confined to unequivocally necessary cases” (Bedford, 1955). Previously, postoperative memory impairments have been reported in 26% of patients aged 60 years and older. These deficits were reported to last months to years by an international multicenter study on postoperative cognitive decline (Moller et al., 1998). Age appears to be the biggest risk factor for changes in cognitive function after surgery (Moller et al., 1998; Monk et al., 2008).

This is concerning because a decline in cognition negatively impacts performance in daily activities (Cahn-Weiner et al., 2000; Owsley and McGwin, 2004; Lee et al., 2005), which results in a loss of independence (Monk et al., 2008) and premature departure from the labor market (Steinmetz et al., 2009). Furthermore, people with postoperative cognitive changes are at higher risk of death in the first year after surgery (Monk and Price, 2011). Over the past 20 years, the number of older patients having surgery has increased faster than the population is aging (Etzioni et al., 2003; Sauër et al., 2009). Therefore, it is important to investigate ways to improve cognitive function in this at-risk population.

Previous studies have reported the benefits of cognitive training programs on cognitive function and mental health in the elderly healthy population as well as the elderly clinical population. There are several types of cognitive training using working memory, processing speed, and video games. These cognitive training programs can improve cognitive performance in memory (Mahncke et al., 2006; Smith et al., 2009), processing speed (Ball et al., 2002, 2007; Edwards et al., 2005), executive function (Bissig and Lustig, 2007; Uchida and Kawashima, 2008), and attention (Mozolic et al., 2011) in the healthy elderly population and the elderly dementia population. Cognitive training also improves mental health in older adults (Iida et al., 2011, 2012; Takeuchi et al., 2014; Nouchi et al., 2016a). There are several reasons why we selected SCRA as the intervention. First, it has been validated in both the healthy elderly population and in the dementia population (Kawashima et al., 2005, 2015; Nouchi et al., 2016b). Second, special machinery and devices are unnecessary to conduct this study, making it very cost-effective (Nouchi et al., 2012b). Third, there is no excessive burden on participants because the time commitment for learning therapy is only 15–30 min each day. Finally, benefits of SCRA were replicated by different cultures (Kawashima et al., 2015).

SCRA intervention consists of reading aloud and solving simple arithmetic, which were specifically chosen based on knowledge of neuroscience. Previously conducted brain imaging studies have indicated that reading aloud (Miura et al., 2003, 2005; Ino et al., 2009; Graves et al., 2010; Parker Jones et al., 2012) and simple math (Menon et al., 2000; Kawashima et al., 2004; Arsalidou and Taylor, 2011) activates the frontal cortex and the association cortices of the temporal and parietal lobes. Therefore, SCRA intervention was specially designed to stimulate these areas, and to consequently promote an improvement in the function of these cortices (Kawashima et al., 2005; Nouchi et al., 2016b).

Previous studies conducted in healthy elderly subjects have demonstrated that SCRA intervention improves executive functioning and processing speed. For example, Uchida and Kawashima (2008) conducted a randomized controlled trial in which subjects were divided into a SCRA intervention group and a waitlisted control group. The SCRA intervention group was instructed to conduct two training tasks 5 days a week: (1) reading Japanese aloud, and (2) conducting simple calculations. After 6-months of intervention, the SCRA intervention group showed improved executive function, which was shown by the frontal assessment battery (FAB) (Dubois et al., 2000; Kugo et al., 2007; Nakaaki et al., 2007), and improved processing speed, as measured by a digit-symbol substitution test (Wechsler, 1997). An examination of the pre- vs. post-test scores for individual items of the FAB also showed that there were statistically significant improvements in the mental flexibility and sensitivity to interference scores of the experimental group, but not in the controls. The researchers concluded that SCRA intervention positively affects some cognitive functions in elderly people (Uchida and Kawashima, 2008). Previous studies have also shown the benefits of SCRA intervention on cognitive functions in healthy elderly adults (Nouchi et al., 2016b) and in elderly adults with dementia (Kawashima et al., 2005). However, it is unclear whether SCRA intervention can lead to improvement of cognitive function and mental health in the elderly postsurgical elderly population. As previously stated, this population has an increased risk of cognitive decline.

### Purpose

The purpose of this study was to investigate the beneficial effects of SCRA intervention on cognitive functions and mental health in the elderly Japanese population after thoracic surgery. We conducted a 12-week open-label randomized control trial with two parallel groups: the SCRA intervention group and wait-listed control group (Kulason et al., 2016). To investigate the positive impact of SCRA intervention on cognitive functions, we utilized a battery of tests such as the Mini-Mental Status Exam-Japanese (MMSE-J) (Pangman et al., 2000), the Frontal Assessment Battery (FAB) (Dubois et al., 2000; Kugo et al., 2007; Nakaaki et al., 2007), and the Cogstate Brief Battery (CBB) (Maruff et al., 2009, 2013; Sauër et al., 2009; Brown et al., 2010; Yoshida et al., 2011; Lim et al., 2012). Additionally, we utilized several mental health questionnaires such as the 12-item General Health Questionnaire-12 (GHQ-12) (Henkel et al., 2003; Jones et al., 2006; Richardson et al., 2007), the Geriatric Depression Scale (GDS) (Yesavage et al., 1982), and the 5-item Quality of Life questionnaire (QOL-5) (Lindholt et al., 2002). These tests and questionnaires were conducted approximately 1 week postoperatively and after SCRA intervention at 3 months postoperatively.

Based on previous studies (Kawashima et al., 2005, 2015; Nouchi et al., 2016b), we expected that SCRA intervention would lead to improvement of cognitive functions. Improvement in executive function was especially expected because SCRA improved executive functions in both the healthy and the clinical elderly populations. In addition, previous cognitive training for young and old healthy adults reported improvement of quality of life and reduction of negative mood (Kwok et al., 2013; Takeuchi et al., 2014; Nouchi et al., 2016a), it was expected that SCRA intervention would improve mental health and reduce depressive moods.

## Methods

### Trial design

**Figure 1:**
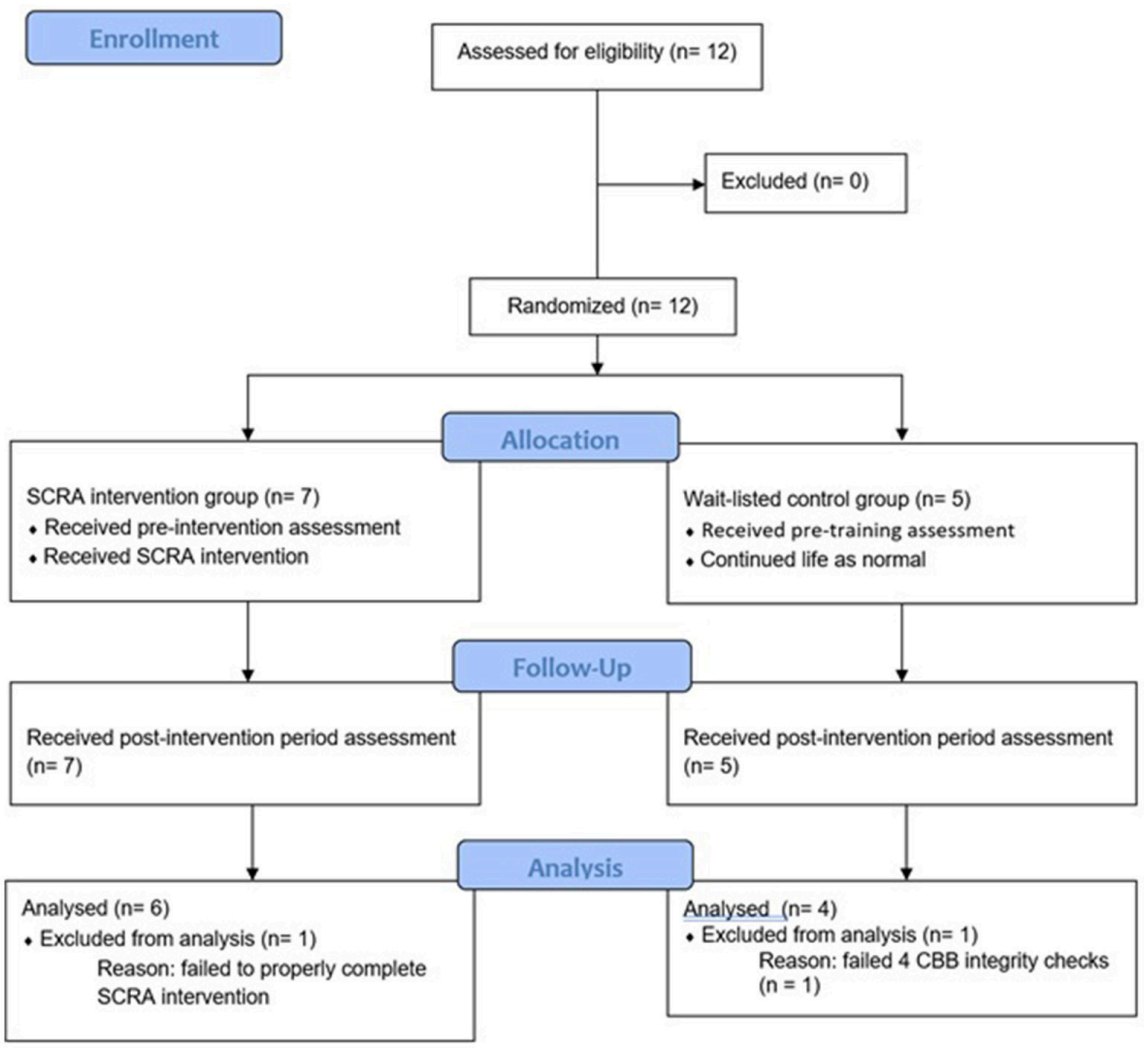
Consolidated standards of reporting trials (CONSORT) flowchart.

The trial protocol for this open-label randomized controlled trial was devised in line with the CONSORT checklist (Supplementary Material 1) for randomized controlled trials (Chan et al., 2013). All subjects who volunteered to participate in this study conducted at Tohoku University Hospital in Sendai city, Miyagi prefecture, Japan, provided written informed consent. The Ethics Committee of Tohoku University Graduate School of Medicine approved both the protocol for this study and the consent form. Additionally, this study was registered with the University Hospital Medical Information Network (UMIN) Clinical Trial Registry (UMIN000019832). The trial design is presented in Figure 1.

### Participants

Twelve pulmonary patients (6 males, 6 females) undergoing non-cardiovascular thoracic surgery were recruited from Tohoku University Hospital. The surgery was conducted using general anesthesia. Participant demographics are noted in Table 1. A doctor of thoracic surgery from the Tohoku University Hospital referred participants to the study over a 3-month period beginning in January 2016. Participants were native Japanese speakers who self-reported to be right-handed and were 60 years and older (mean age 70.16 ±6.07 years). Participants were not particularly concerned with potential changes in their memory and cognition. A total of two participants were removed from all analysis. One participant was removed from the wait-listed control group for failing 4 CCB integrity checks over the two testing sessions. The other participant was removed for failing to properly complete the SCRA intervention.

**Table 1:** Demographic information.

**GENDER**	
Treatment	3 males, 3 females
Control	1 males, 3 females
**MEAN AGE (yrs.)**	
Treatment	69 ± 6.96
Control	68.75 ± 4.27
Race/Ethnicity	Japanese
Procedure	Partial pulmonary lobectomy
Location of procedure	Tohoku University Hospital
Mean anesthesia duration (min)	257.45 ± 70.14
**MEAN ANESTHESIA ADMINISTERED**	
Remifentanil (mg)	4.31 ± 2.47
Fentanyl (mg)	0.28 ± 0.13

Patients admitted to the hospital were informed about the study prior to surgery. Interested patients received both a written and a verbal explanation of the study. Prior to participating in the study, all subjects were requested to sign the informed consent form. There were no significant differences between the groups in all data (two-sample *t*-test, *p* > 0.10). MMSE scores were within the normal range.

### SCRA intervention group

**Figure 2:**
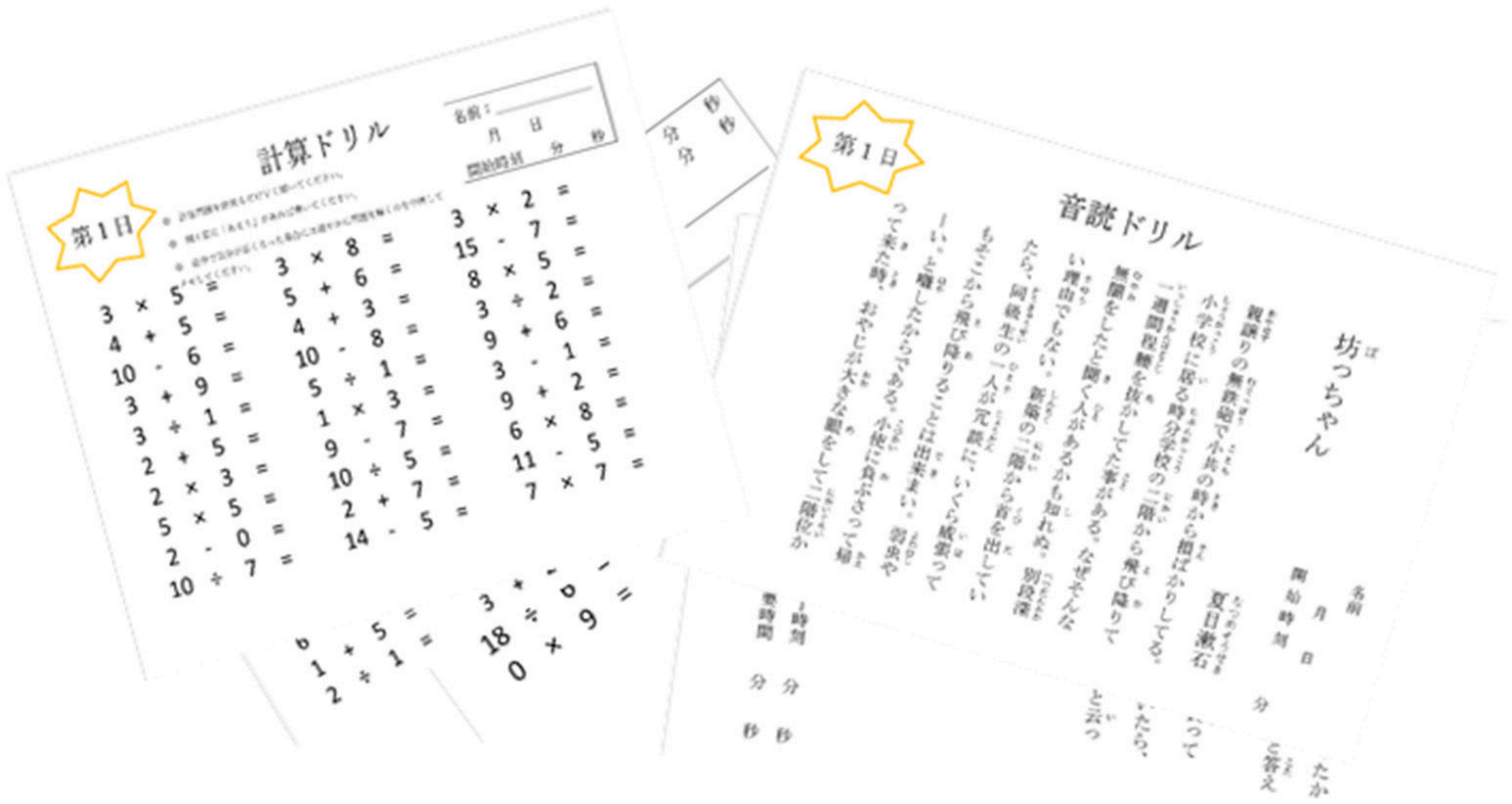
Sample simple calculation and reading aloud (SCRA) cognitive intervention. Sixty days of arithmetic **(Left)** and reading aloud **(Right)** worksheets similar to those depicted above were given to subjects randomly placed into the intervention group.

Subjects were randomly placed into the SCRA intervention group (*n* = 6). The 30-min SCRA intervention (Figure 2) was conducted 3–5 times a week for 12 weeks. The cognitive intervention material were prepared from Dr. Ryuta Kawashima's published book series “Training the Brain: The Adult's Arithmetic Drills” 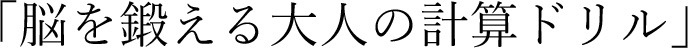 and “Training the Brain: The Adult's Verbal Reading Drills” 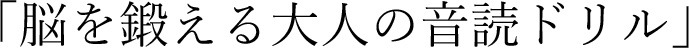, which have already been shown to be effective (Kawashima et al., 2004; Uchida and Kawashima, 2008). Study subjects received the intervention packet before being discharged from the hospital. Upon receiving verbal and written instruction for the intervention, participants were given a practice set of worksheets to ensure comprehension.

The difficulty level for arithmetic materials ranged from single-digit addition to double-digit division. The first 10 days of the arithmetic worksheets consisted of 50 problems, and the remaining 50 days consisted of 100 problems. Participants were instructed to complete the arithmetic as quickly and accurately as possible, and to stop after 15 min even if they did not reach the end. Participants recorded the time it took to complete each worksheet with the provided timer, and project investigators graded the accuracy for all completed arithmetic worksheets. The reading worksheets were 1–2 pages of short stories and essays. Subjects were instructed to read the text aloud and to enunciate each word. The time it took to complete each worksheet was also recorded. Again, the participant was instructed to stop if they exceeded 15 min.

Participants were given the three cognitive assessments (MMSE-J, FAB, CBB) and the three psychological questionnaires (GHQ-12, GDS, QOL-5) detailed above before starting the intervention, and after completing the intervention.

### Waitlisted control group

During the duration of the intervention, the waitlisted control group (*n* = 4, 2 excluded) continued life as usual. They did not receive SCRA cognitive intervention. After the conclusion of the study, the waitlisted subjects were offered the opportunity to experience SCRA intervention. Intervention studies conducted previously (Clark et al., 1997; Mahncke et al., 2006) have reported that there is no need for a placebo group for this type of study because there are no differences between the placebo group and control groups regarding improvement in cognitive function. Participants were given the three cognitive assessments (MMSE-J, FAB, CBB) and the three psychological questionnaires (GHQ-12, GDS, QOL-5) detailed above before starting the intervention, and after completing the intervention.

### Cognitive function outcome measures

A total of three cognitive tests batteries were administered to participants at two time points. Baseline was administered postoperatively (7.7 ± 3.06 days), and follow-up testing was administered after completing the 3-month SCRA intervention. All tests were administered by trained project investigators. Additionally recorded data included the type, amount, and duration of general anesthesia, surgery duration, age, and sex. The first cognitive test battery was the Mini-Mental State Examination-Japanese (MMSE-J), a 30-point cognitive test used extensively in both research and clinically to measure cognition (Pangman et al., 2000; Sugishita, 2012). The higher the MMSE-J score, the better the cognitive performance. In research on postoperative cognitive decline, the MMSE is one of the most common assessments used to determine mental status (Folstein et al., 1975; Wang et al., 2014). The MMSE has a test-retest interval of 2 months (Folstein et al., 1975; Tombaugh and McIntyre, 1992; Helkala et al., 2002).

The second cognitive test battery was the FAB, an 18-point cognitive assessment which can be performed at the bedside or in a clinical setting to measure executive functions. There are six subsets to the FAB which explore different functions of the frontal lobe: conceptualization, mental flexibility, motor programming, sensitivity to interference, inhibitory control, and environmental autonomy (Dubois et al., 2000; Kugo et al., 2007; Nakaaki et al., 2007). Higher scores indicate better performance. The Japanese version of the FAB is comparable, in terms of validity and reliability, to the original English version (Kugo et al., 2007; Nakaaki et al., 2007). The FAB is commonly administered in conjunction with the MMSE in studies examining cognitive decline (Brown et al., 2010; Nouchi et al., 2012a; Bugalho et al., 2013; Barulli et al., 2015).

Finally, participants were tested using a laptop running the CBB to measure the speed of processing, visual attention, visual learning and memory, and attention and working memory. The CBB, which consists of four tasks, has been shown to effectively determine cognitive decline (Maruff et al., 2009, 2013; Sauër et al., 2009; Brown et al., 2010; Lim et al., 2012) and has also been shown to be effective in the Japanese population (Yoshida et al., 2011). As a computerized test, CBB is designed to have virtually an unlimited number of variations. There are no retest-related effects in CBB scores after 1 month; however, there are re-test effects at 1-week (Maruff et al., 2009, 2013; Lim et al., 2012). A series of internal integrity checks were applied to the CBB data to ensure that each subject was completing each task properly. The four CBB tasks have previously been described in detail (Collie et al., 2002; Falleti et al., 2006; Maruff et al., 2013) and on the Cogstate website (https://cogstate.com/). The tests are briefly summarized here.

Administered first was the detection (DET) task, which measures psychomotor function. Since the primary performance measure is normalized using a log_10_ transformation, a lower score indicates better performance. The next task, identification (IDN) measured visual attention. Again, the primary performance measure was normalized using a log_10_ transformation, and therefore, a lower score indicates better performance. Administered third was the one card learning (OCL) task, which is a continuous visual recognition learning task that assesses visual learning within a pattern separation model (Yassa et al., 2010). The proportion of correct answers was recorded and normalized using an arcsine square-root transformation; therefore, a lower score indicates worse performance. Administered last was the one-back (OBK) task, which assesses working memory and attention by recording the proportion of correct answers. The primary performance measure was normalized using an arcsine square-root transformation, and a lower score indicates worse performance.

### Psychological questionnaires outcome measures

Immediately after the cognitive assessments before and after the intervention period, three psychological questionnaires were administered to measure mental and emotional state: GHQ-12, GDS, and QOL-5. All questionnaires are self-reported measures. The GHQ-12 assesses psychological morbidity and is extensively used in clinical practice (Richardson et al., 2007), epidemiological research (Henkel et al., 2003), and psychological research (Jones et al., 2006) to screen the domains of depression, anxiety, somatic symptoms, and social withdrawal. A higher score indicates that the individual is at higher risk of developing a psychiatric disorder. The GDS screens for depression in the elderly population by assessing cognitive and emotional symptoms of depression (i.e., feelings of worthlessness, preference for staying at home, concern about memory problems), and a higher score indicates higher psychological distress (Yesavage et al., 1982). The QOL-5 measures global and generic quality of life that has been shown to have internal consistency and sensitivity and relevance. It also has practical outcome measures that are available for clinical databases (Lindholt et al., 2002). A higher score indicates a greater quality of life.

### Sample size

This was a pilot study, and similar studies have not been previously conducted. Therefore, it was not possible to calculate a sample size. Future studies can calculate the sample size based on the pilot study.

### Randomization and blinding

Participants were randomly allocated into one of two groups: the SCRA cognitive intervention group, and the wait-listed control group. The project investigator (RN) used an online computer program (http://www.graphpad.com/quickcalcs/index.cfm) to assign participants their group designation. Participants were stratified by gender, and were blocked randomized (block size; 4) with an allocation ratio of 1:1 to create similar size groups. It is difficult to design a fully blinded study in a design in which one group receives an intervention and the other receives nothing. As a result, the trial designed was open- label.

### Statistical analysis

This study was designed to evaluate the effect of SCRA cognitive intervention in elderly Japanese patients who have undergone lung surgery. CBB scores were normalized around 100 with a standard deviation of 10. To check the beneficial effects of SCRA, we conducted the following group difference and within-group analyses. First, to check group differences, change in score was calculated (post—pre) for each outcome measure. The sign for the change in score of DET and IDN were reversed so that negative numbers would reflect worse performance. An ANCOVA with permutation test was conducted to determine significant differences in scores between the intervention group, the wait-listed control group, and the covariates (baseline score, age, sex). We used the permutation test for ANCOVA models for two reasons. First, it is suited toward small sample analysis with no restrictions on distribution (May and Hunter, 1993; Ludbrook and Dudley, 1998; Anderson, 2001; Kherad-Pajouh and Renaud, 2010). Second, the permutation test corrects Type 1 error (false positive) (Shuster and Boyett, 1979; Anyela et al., 2008).

For within-group analysis, a Wilcoxon signed-rank test was employed to determine significant changes in within-group pre-intervention scores and post-intervention scores. We used one-tailed ANCOVAs and Wilcoxon signed-rank tests because we had a strong hypothesis that the SCRA intervention would improve cognitive functions and emotional states. The level of significance was set at *p* < 0.05. We did not use any multiple comparison methods to adjust *p*-values (i.e., FDR) because this is a pilot study with a small sample size. Several methods are available (i.e., multiple testing corrections and re-sampling) to control Type 1 error. The Bonferroni (Hsu, 1996) and Benjamini and Hochberg (False discovery rate; FDR) (Benjamini and Hochberg, 1995) are two such correction methods that are commonly used. However, the While the Bonferroni correction is very conservative, it can create unacceptable levels of Type II errors and contribute to publication bias, or the exclusion of a potentially relevant hypothesis (Nakagawa, 2004). On the other hand, the FDR method is less stringent, but may lead to the acceptance of a false positive hypothesis. Therefore, permutation tests, a typical resampling method (Belmonte and Yurgelun-Todd, 2001), are now widely accepted and recommended for multiple statistical testing (Shuster and Boyett, 1979; Nakagawa, 2004; Anyela et al., 2008; Nouchi et al., 2013). Statistical analysis was conducted using RStudio [version 3.2.4 (2016-03-10)].

## Results

All participants scored similarly on all outcome measures at baseline. There were no significant differences at baseline between the wait-listed control group scores and the SCRA intervention group scores (MMSE *p* = 0.645; FAB *p* = 0.412; DET *p* = 0.457; IDN *p* = 0.369; OCL *p* = 0.070; OBK *p* = 0.226; GDS *p* = 0.060; GHQ *p* = 0.253; QOL *p* = 0.224).

### Between-group analyses

To check the benefits of SCRA on cognitive function and mental health compared to the control group, we conducted an ANCOVA with permutation test examining change in score (Table 2) between the intervention group and the wait-listed control group. The analysis determined that the intervention group had a significant improvement the FAB motor programming sub-score compared to the control group (*p* = 0.0195, **Table 4**). There was no significant difference in between group change in total FAB scores (Table 3); however, the intervention group's total FAB scores appear to have a generally positive trend. There was also a significant improvement in the intervention group GDS score compared to that of the control group (*p* = 0.0259; Table 3). Additionally, there was a significant improvement in the intervention group QOL5 score compared to the control group (*p* = 0.038; Table 3). There were no other significant differences in the change in scores between the two groups. These results clearly show that the SCRA intervention improves motor programming, depressive mood, and quality of life compared to the control group.

**Table 2:** Change in primary outcome measure scores.

**Subject**	**ΔMMSE**	**ΔFAB**							**ΔDET**	**ΔIDN**	**ΔOCL**	**ΔOBK**	**ΔGDS**	**ΔGHQ**	**ΔQOL**
			**Δconcept**	**Δmental**	**Δmotor**	**Δinterfer**.	**Δinhib. ctrl**.	**Δenvir. auto**.							
**WAITLIST CONTROLS**															
Sub3	−1	−2	0	0	−2	0	0	0	3	8	14	2	5	4	−5
Sub4	−3	−2	−1	0	0	0	−1	0	−4	−4	6	17	12	0	−12
Sub5	0	2	0	1	0	3	−2	0	0	2	4	16	−1	4	5
Sub7	2	4	0	0	3	1	0	0	6	8	−4	−6	0	0	0
**SCRA INTERVENTION**															
Sub1	1	2	1	0	2	0	−1	0	4	−2	8	0	0	0	4
Sub2	1	1	0	1	0	0	0	0	12	0	4	13	−1	1	3
Sub6	−1	3	0	1	2	0	0	0	−5	−6	1	0	0	4	6
Sub8	1	3	0	0	3	0	0	0	−6	−6	−12	18	1	−1	−2
Sub9	−9	1	0	0	0	0	0	0	18	9	−2	5	−2	−2	0
Sub11	−1	1	0	0	1	0	0	0	−9	−13	6	−13	−3	1	9

*All values were calculated (Post-intervention—Pre-intervention). The sign for DET, IDN, and GHQ12 were reversed so that a negative number reflects worse performance. Therefore, all negative values in this table indicate a decline. FAB sub-score measures include concept (Δconcept), mental flexibility (Δmental), motor programming (Δmotor), interference (Δinterfer.), inhibitory control (Δinhib. ctrl.), and environmental autonomy (Δenvir. auto.)*.

**Table 3:** Primary outcome measure scores.

	**Basline mean (±*SD*)**	**Baseline range**	**Follow-up mean (±*SD*)**	**Follow-up range**	**Change in score**	**Within group *p*-value**	**Between group *p*-value**
MMSE intervention	28.33 (±1.37)	26 to 30	27 (±5.02)	17 to 30	−0.05 (±0.15)	0.3695	0.5^#^
MMSE control	28.75 (±0.5)	28 to 29	28.25 (±1.71)	26 to 30	−0.02 (±0.07)	0.2965	
FAB intervention	13.33 (±1.51)	12 to 15	15.17 (±2.04)	13 to 17	0.14 (±0.07)	0.013^*^	0.5^#^
FAB control	13 (±1.63)	11 to 15	13.7 (±2.04)	9 to 17	0.04 (±0.23)	0.3525	
DET intervention	95.17 (±5.88)	86 to 103	92.83 (±10.17)	75 to 102	−0.02 (±1.12)	0.422	0.5^#^
DET control	95 (±6.98)	87 to 103	93.75 (±10.69)	81 to 107	−0.02 (±0.05)	0.2965	
IDN intervention	101.5 (±1.22)	100 to 103	98.17 (±7.39)	84 to 105	−0.03 (±0.07)	0.138	0.5^#^
IDN control	100.25 (±3.86)	87 to 103	96.75 (±8.77)	95 to 108	−0.04 (±0.06)	0.1345	
OCL intervention	102.17 (±5.46)	92 to 106	103 (±5.48)	94 to 106	0.01 (±0.07)	0.344	0.5^#^
OCL control	97.5 (±3.42)	94 to 102	102.5 (±5.74)	98 to 110	0.05 (±0.07)	0.099	
OBK intervention	106.33 (±8.21)	98 to 116	110.17 (±6.55)	103 to 116	0.04 (±0.10)	0.1785	0.5^#^
OBK control	100.75 (±1.71)	99 to 103	108 (±9.56)	97 to 116	0.07 (±0.11)	0.1875	
GHQ12 intervention	1.67 (±1.51)	0 to 4	1.17 (±0.98)	0 to 2	−0.5 (±2.07)	0.34	0.235
GHQ12 control	2.75 (±2.63)	0 to 5	0.75 (0.50)	0 to 1	−2 (2.32)	0.0785	
GDS intervention	4.12 (±2.14)	0 to 6	3.33 (±2.34)	0 to 6	−0.83 (±1.47)	0.099	0.0259^*^
GDS control	1.5 (±1.00)	1 to 3	5.5 (±6.86)	0 to 15	4 (±5.94)	0.1425	
QOL5 intervention	14.5 (±3.56)	9 to 17	17.83 (±2.93)	15 to 23	3.33 (±3.98)	0.0398^*^	0.038^*^
QOL5 control	16.75 (±3.10)	14 to 21	13.75 (±8.14)	3 to 21	−3 (±7.26)	0.207	

*Note that DET and IDN measure reaction time in milliseconds, so higher scores indicate poorer performance. A higher score indicates better performance for all other test scores (OCL, OBK, MMSE, FAB). A higher GHQ12 score indicates poorer overall psychiatric health, and a higher score for GDS and QOL5 indicate better psychiatric health. Within-group analysis conducted by Wilcoxon signed-rank test, and between-group analysis conducted by ANCOVA with permutation test. There was a significant improvement in the total FAB scores within the intervention group (p = 0.013), but not in controls (p = 0.353). There was also a significant improvement in the intervention GDS scores compared to controls (0.0259). Additionally, there was a significant improvement in the intervention groups QOL5 score compared to controls (p = 0.038), as well as a significant improvement within the intervention group comparing baseline to follow-up scores (p = 0.0398) and not within the controls group (p = 0.207)*.

* *The significance level was set at p < 0.05*.

# *The statistical value is close to zero which resulted in a two-tailed p-value of 1.0 (1-tail p-value is 0.5)*.

### Within-group analyses

To investigate how cognitive function and mental health changed between baseline and follow-up within each group, an analysis of within-group score changes were conducted using a Wilcoxon signed-rank test. There was a significant improvement in the FAB motor programming sub-score within the intervention group in the follow-up score compared to baseline (*p* = 0.0095, Table 4), but not in controls (*p* = 0.376, Table 4). Additionally, there was an improvement in the total FAB scores within the intervention group in the follow-up score compared to baseline (*p* = 0.013, Table 3), but not in controls (*p* = 0.353, Table 3). Additionally, the intervention group QOL5 score significantly improved after the intervention (*p* = 0.0398, Table 3), but not in the control group (*p* = 0.207, Table 3). There were no other significant changes in scores within each group.

**Table 4:** FAB sub-score outcome measures.

		**Basline mean (±*SD*)**	**Baseline range**	**Follow–up mean (±*SD*)**	**Follow–up range**	**Change in score**	**Within group *p-*value**	**Between group *p*-value**
Concept	Intervention	2.17(±0.41)	2–3	2.33 (±0.52)	2–3	0.08 (±0.20)	0.1585	0.5^#^
	Control	2.5 (±0.58)	2–3	2.25 (±0.5)	2–3	−0.08 (±0.17)	0.1585	
Mental Flexibility	Intervention	2.17 (±0.75)	1–3	2.5 (±0.84)	1–3	0.125 (±0.25)	0.0785	0.5^#^
	Control	2.5 (±0.58)	2–3	2.75 (±0.26)	2–3	0.17 (±0.26)	0.1585	
Motor programming	Intervention	0.83 (±0.41)	0–1	2.17 (±0.98)	1–3	0.83 (±0.98)	0.0095^*^	0.0195^*^
	Control	1.25 (±1.5)	0–3	1.5 (±1.73)	0–3	−0.25 (±0.5)	0.376	
Interferance	Intervention	2.83 (±0.41)	2–3	2.83 (±0.41)	2–3	0 (±0.00)	0.5^#^	0.5^#^
	Control	1.25 (±1.5)	0–3	2.5 (±1)	1–3	0.125 (±0.25)	0.108	
Inhibtory control	Intervention	2.33 (±1.03)	1–3	2.17 (±0.98)	1–3	−0.06 (±0.14)	0.3575	0.5^#^
	Control	2.25 (±0.96)	1–3	1.5 (±1)	1–3	−0.29 (±0.34)	0.134	
Environmental autonomy	Intervention	3 (±0.00)	3–3	3 (±0.00)	3–3	0 (±0.00)	NA	0.5^#^
	Control	3 (±0.00)	3–3	3 (±0.00)	3–3	0 (±0.00)	NA	

*The FAB consists of six sub-score measures: concept, mental flexibility, motor programming, interference, inhibitory control, and environmental autonomy. Within-group analysis conducted by Wilcoxon signed-rank test, and between-group analysis conducted by ANCOVA with permutation test. There was a significant improvement in the intervention group FAB motor programming sub-score compared to controls (0.0195). There was also a significant improvement in the FAB motor programming sub-score within the intervention group comparing baseline to follow-up (0.0095), and not in the control group (n = 0386)*.

* *The significance level was set at p < 0.05*.

# *The statistical value is close to zero which resulted in a two-tailed p-value of 1.0 (1-tail p-value is 0.5)*.

## Discussion

This pilot study investigated the benefits of SCRA intervention in an elderly Japanese postsurgical population. To reiterate, age has been identified as a major risk factor for changes in cognition after surgery (Moller et al., 1998). This was the first study conducted in the elderly Japanese post-thoracic surgical population that investigated the effects of a non-invasive paper-and-pencil intervention based on learning therapy. The preliminary results show that the SCRA intervention improves motor programming, symptoms of depressive moods, and quality of life.

The first main finding is a significant improvement in FAB motor programming sub-scores after the intervention in the experimental group compared to the control group. Additionally, the FAB motor programming sub-scores within the intervention group significantly increased after the intervention. An increase in FAB motor programming sub-scores was not present in the control group. This suggests that SCRA intervention improves motor programming. Motor programming is a term used to represent the movement that centrally organizes and controls the degrees of freedom involved in movement. It encompasses the signals transmitted through the efferent and afferent pathways of the central nervous system that enables the brain to anticipate, plan, and guide movement (Bastian, 2008). Patients with lesions in their frontal lobe, which is heavily involved in motor programming, may have impairments in tasks that require temporal organization, maintenance, and execution of successive actions—all of which are necessary for motor programming (Milner, 1984; Jason, 1986; Dubois et al., 2000). The preliminary data also found that the total FAB score within the intervention group significantly increased post-intervention but did not increase in the control group. A significant difference between the two groups was not present in the change in total FAB scores. This may be due to the small sample size. The preliminary data is not sufficient to draw any conclusions, and further research with a larger subject pool is necessary.

In this present study, the intervention involved the use of paper and pencil (reading aloud and simple arithmetic). It is possible that the frequent use of fine motor functions during the intervention contributed to the observed increase in FAB motor programming sub-scores. Additionally, improvements in motor programming can be explained by the overlapping hypothesis (Nouchi and Kawashima, 2014; Nouchi et al., 2014, 2016b). The hypothesis makes an assumption that cognitive functions are improved by cognitive training given that the training and the affected cognitive functions involve the same brain areas. In this study, participants conducted simple arithmetic and reading aloud training tasks, which involves areas in the frontal, temporal, and parietal cortices. Based on the overlapping hypothesis, the improvements in motor programming is because the training task and motor programming share similar mental processes. Interestingly, the motor programming task has been found to activate the prefrontal cortex more than other tasks including inhibitory control tasks (Toyoda et al., 2016). Therefore, conducting the SCRA intervention would activate mental processes involved in motor programming—the frontal cortex. As a result, there was an improvement in motor programming due to the intervention.

While the FAB motor programming sub-score does appear to significantly improve in the intervention group, the lack of improvement in other cognitive measures in addition to the lack of improvement in the total FAB score does not reflect the beneficial effects of learning therapy observed by both Uchida and Kawashima (2008) and Kawashima et al. (2005). As previously mentioned, Uchida and Kawashima (2008) examined change in baseline and follow-up FAB sub-scores within each group found statistically significant improvements in mental flexibility and in conflicting instruction scores of the experimental group, but not in the controls. However, there are several key differences between this present study and Uchida and Kawashima (2008). First, the intervention period for their study was 6-months, and ours was only 3-months. Second, the intervention for their study was more intensive in that subjects were required to go to a learning center 1 day a week and to do their homework for 4 days a week. The SCRA intervention was laxer since the requirement was to do the training material 3–5 times a week at home. Third, the age range of the subjects for their study was 70–85 years old (mean 75.3 ±3.8). The age range for our study was 60–79 years old (mean 70.16 ±6.07). It is possible that the lower intervention frequency and shorter intervention period for this present study were not enough to improve mental flexibility and conflict. Additionally, our subjects were generally younger than the subjects in Uchida and Kawashima (2008), and therefore there may have been less room for dramatic improvement in mental flexibility and conflict. Since this is a pilot study, clear conclusions cannot be drawn and further research is necessary.

The second main finding of this preliminary data is that SCRA reduced the GDS scores. Lower GDS indicates lower psychological distress. Therefore, the intervention group suggests that SCRA intervention helped reduce cognitive and emotional symptoms of depression. Our preliminary findings also indicated a significant improvement in the QOL5 score of the intervention group compared to the controls. A higher QOL5 score reflects a higher quality of life. Therefore, the significantly greater positive change in the intervention group QOL5 score compared to that of the control group suggests that the SCRA intervention helped improve quality of life. The QOL5 score of the intervention group was also significantly greater after the intervention. This significant improvement in QOL5 scores was not present in the control group. These results further support the idea that SCRA intervention improves quality of life.

It is possible that the SCRA intervention functioned as an emotional regulator. Conducting the intervention may have served to divert a person's attention away from negative emotional experiences, and helped a person to ignore negative experiences. In fact, it has been reported that cognitive activity can modulate subsequent psychological and physiological emotional processes (Iida et al., 2011, 2012). There have also been several studies in which cognitive training for working memory and processing speed reduce negative emotion and depressive symptoms (Takeuchi et al., 2014; Nouchi et al., 2016a). It is important to note that speed of processing involves the parietal and temporal cortices (Turken et al., 2008). As previously mentioned, the SCRA intervention trains the frontal cortex and of the temporal and parietal association cortices, and therefore this preliminary finding of this present study agrees with previous findings.

This pilot study has several major limitations. First, the waitlisted control group was not given a placebo because it would be unreasonable to ask elderly patients recovering from surgery to complete tasks designed to have zero effect. However, the lack of a placebo may introduce a performance bias and a difference in subject motivation to completing the testing measures. Nevertheless, studies have indicated that a placebo for the control group is not required for this type of study (Clark et al., 1997; Mahncke et al., 2006). The purpose of this study is to determine the effects of SCRA intervention in the postoperative elderly population. Although the waitlisted control group is sufficient, in the future it may be necessary to use an active control group.

The second limitation is the small sample size. Based on this pilot study, there is a real need for this current study to be conducted with larger sample sizes to yield more conclusive results. Future studies may also benefit from examining the effects of SCRA intervention in patients undergoing different surgical procedures with a wide range of anesthetic type and duration. Third, the literature suggests that brain training only affects domains that are specifically trained, and only for the duration of the training (Rowe and Kahn, 1997; Ball et al., 2002; Franklin et al., 2003; Günther et al., 2003; Klingberg et al., 2005; Sartory et al., 2005; Salthouse, 2006; Sammer et al., 2006; Sitzer et al., 2006; Willis et al., 2006; Schooler, 2007; Dahlin et al., 2008; Barnes et al., 2009; Fisher et al., 2009; Jaeggi et al., 2011; Kueider et al., 2012; Woods et al., 2012; Melby-Lervåg and Hulme, 2013; Ahmed et al., 2015; Bell et al., 2016). Therefore, future studies on improving cognitive functions in this population may see more prominent results if the treatment is tailored toward improving what is measured (i.e., processing speed, visual attention). Nevertheless, the preliminary results together suggest that SCRA intervention improves motor programming, and possibly improve general frontal lobe functions.

In summary, the use of SCRA intervention significantly improved motor programming functions of the frontal lobe. The intervention also significantly improved symptoms of depressive mood and quality of life. Further research is necessary before any clear conclusions can be drawn. However, this pilot study provides a solid foundation and direction for future studies. Exploring the relationship between postoperative training motor programming and depressive moods and quality of life may be beneficial for future postoperative elderly patients. Another potential direction for future research is to explore preoperative cognitive training. One randomized control trial reports that patients preoperatively trained in a cognition mnemonic skill for a total of three 1-h sessions with the method of *loci* significantly lowered the incidence of postoperative cognitive decline in the intervention group compared to the controls 1 week after surgery (Saleh et al., 2015). Further research on the effects of preoperative motor programming training on cognitive change, emotional well-being, and quality of life is warranted.

## Protocol

The full trial protocol can be found at Kulason et al. (2016).

## Author contributions

KK, RN, YH, MN, YO, and RK designed developed the study protocol; KK and RN searched the literature, selected cognitive function measures, created manuals to conduct, and rate cognitive measures; KK and RN wrote the manuscript with YH, MN, YO, and RK; RK also gave advice related to the study protocol. All authors read and approved the final manuscript.

### Conflict of interest statement

Learning therapy was developed by RK and KUMON Institute of Education. However, RK derives no income from KUMON Institute of Education and Society for Learning Therapy. The other authors declare that the research was conducted in the absence of any commercial or financial relationships that could be construed as a potential conflict of interest.

## Abbreviations

ANCOVA: analysis of covariance
CONSORT: consolidated standards of reporting trials
RCT: randomized controlled trial
MMSE: mini-mental status exam
FAB: frontal assessment battery
SCRA: simple calculation and reading aloud
SPSS: statistical package for the social sciences
UMIN: University Hospital Medical Information Network.
